# Human umbilical cord-derived mesenchymal stem cells improve the function of liver in rats with acute-on-chronic liver failure via downregulating Notch and Stat1/Stat3 signaling

**DOI:** 10.1186/s13287-021-02468-6

**Published:** 2021-07-13

**Authors:** Yulin He, Xingrong Guo, Tingyu Lan, Jianbo Xia, Jinsong Wang, Bei Li, Chunyan Peng, Yue Chen, Xiang Hu, Zhongji Meng

**Affiliations:** 1 Institute of Biomedical Research, Hubei Clinical Research Center for Precise Diagnosis and Treatment of Liver Cancer, Taihe Hospital, Hubei University of Medicine, Shiyan, 442000 Hubei China; 2 Hubei Key Laboratory of Embryonic Stem Cell Research, Shiyan, 442000 Hubei China; 3 Postgraduate Training Basement of Jinzhou Medical University, Taihe Hospital, Hubei University of Medicine, Shiyan, 442000 Hubei China; 4 Department of Infectious Diseases, Taihe Hospital, Hubei University of Medicine, Shiyan, 442000 Hubei China; 5 Department of Laboratory Medicine, Maternal and Child Health Hospital of Hubei Province, Wuhan, 430070 Hubei China; 6 Shenzhen Beike Biotechnology Research Institute, Nanshan District, Shenzhen, 518057 China

**Keywords:** Liver fibrosis, Liver injury, ACLF, hUC-MSCs, Paracrine, Notch, Stat1, Stat3

## Abstract

**Background:**

Effective treatments for acute-on-chronic liver failure (ACLF) are lacking. Human umbilical cord-derived mesenchymal stem cells (hUC-MSCs) have been applied in tissue regeneration and repair, acting through paracrine effects, cell fusion, and actual transdifferentiation. The present study was designed to investigate the therapeutic potential of hUC-MSCs in acute-on-chronic liver injury (ACLI) and ACLF rat models.

**Methods:**

Wistar rats aged 6 weeks were intraperitoneally administered porcine serum (PS) at a dose of 0.5 mL twice per week for 11 weeks to generate an immune liver fibrosis model. After 11 weeks, rats with immune liver fibrosis were injected intravenously with lipopolysaccharide (LPS) to induce an ACLI model or combined LPS and D-galactosamine (D-GalN) to induce an ACLF model. The rats with ACLI or ACLF were injected intravenously with 2×10^6^ hUC-MSCs, 4×10^6^ hUC-MSCs, or 0.9% sodium chloride as a control. The rats were sacrificed at 1, 2, 4, and 6 weeks (ACLI rats) or 4, 12, and 24 h (ACLF rats). The blood and liver tissues were collected for biochemical and histological investigation.

**Results:**

The application of hUC-MSCs in rats with ACLI and ACLF led to a significant decrease in the serum levels of ALT, AST, TBil, DBil, ALP, ammonia, and PT, with ALB gradually returned to normal levels. Inflammatory cell infiltration and collagen fiber deposition in liver tissues were significantly attenuated in ACLI rats that received hUC-MSCs. Inflammatory cell infiltration and apoptosis in liver tissues of ACLF rats that received hUC-MSCs were significantly attenuated. Compared with those in the rats that received 0.9% sodium chloride, a significant reduction in proinflammatory cytokine levels and elevated serum levels of hepatocyte growth factor (HGF) were found in ACLF rats that received hUC-MSCs. Furthermore, Notch, IFN-γ/Stat1, and IL-6/Stat3 signaling were inhibited in ACLI/ACLF rats that received hUC-MSCs.

**Conclusions:**

hUC-MSC transplantation can improve liver function, the degree of fibrosis, and liver damage and promote liver repair in rats with ACLI or ACLF, mediated most likely by inhibiting Notch signaling and reversing the imbalance of the Stat1/Stat3 pathway.

**Supplementary Information:**

The online version contains supplementary material available at 10.1186/s13287-021-02468-6.

## Background

Chronic liver disease (CLD) refers to a type of disease with a history of cirrhosis or noncirrhosis of the liver for more than 6 months. According to the latest global disease burden research data, 1.5 billion people worldwide suffered from CLD in 2017, the most common types of which are nonalcoholic fatty liver disease (NAFLD), viral hepatitis, and alcoholic liver disease [1]. Acute-on-chronic liver injury (ACLI) is an acute liver injury caused by hepatitis virus, superinfection, alcohol consumption, ischemia and hypoxia, drugs, or immune damage on the basis of CLD. ACLI is a common disease in the clinic, but the disease progresses rapidly in some patients and develops into acute-on-chronic liver failure (ACLF). ACLF is a clinical syndrome characterized by severe jaundice and coagulation dysfunction that combine with complications, such as hepatic encephalopathy, ascites, electrolyte disturbance, infection, hepatorenal syndrome, and hepatopulmonary syndrome. The mortality of ACLF reaches 50–80% [2, 3]. The current treatment of ACLI and ACLF is mainly limited to supportive treatment, and there is no specific therapy. Artificial liver treatment plays an important role, but its clinical application is limited due to the shortage of blood supplies, infections, bleeding, and other complications [4, 5]. Liver transplantation is a radical cure operation, but its clinical application is limited by the shortage of donor livers, adverse postoperative reactions, and high medical expenses [6, 7]. Therefore, it is urgent to explore new treatment options to improve the quality of life and survival of patients with ACLI and ACLF.

Bacterial /virus infection or acute alcoholic hepatitis that occurs on the basis of CLD can induce innate immunity or an acquired immune response and induce systemic inflammation (SI). In severe cases, it progresses to systemic inflammatory response syndrome (SIRS), and the more severe the SIRS is, the worse the prognosis [8]. Serum levels of the proinflammatory cytokines interleukin-1 (IL-1), interleukin-6 (IL-6), tumor necrosis factor-α (TNF-α), and transforming growth factor-β (TGF-β) in patients with hepatitis B virus (HBV)-related ACLI and ACLF are significantly increased, especially those of IL-6, TNF-α, and TGF-β, which are significantly higher in patients with ACLF than in those without ACLF [9]. The white blood cell count and c-reactive protein level in ACLF patients are significantly higher than those in patients without ACLF, and the white blood cell count and c-reactive protein level are positively correlated with the number of organ failure and the severity of the disease in ACLF patients, suggesting that SI may account for the pathogenesis of ACLF in patients with decompensated liver cirrhosis [10]. SI is the main driving factor for the occurrence and development of ACLF and is closely related to the occurrence and development of extrahepatic organ failure, such as hepatic encephalopathy, renal failure, lung failure, and circulatory failure. As the disease progresses, the immune status of ACLF patients evolves from a proinflammatory state to an antiinflammatory state. SIRS and compensatory antiinflammatory response syndrome (CARS) jointly drive the occurrence and development of ACLF and multiple organ failure [11]. A study confirmed that the histological characteristics of HBV-ACLF are sublarge liver necrosis with obvious inflammatory cell infiltration in the necrotic liver tissue, and the level of antiinflammatory cytokines, such as interleukin-10 (IL-10), in patients with sublarge liver necrosis is obviously increased, proving that the SI of ACLF patients changes with disease progression [12]. With ACLF progression, the inflammatory response balance gradually shifts from a proinflammatory response to an antiinflammatory response. The antiinflammatory response dominates in ACLF at the late stage, namely, CARS, and the production of antiinflammatory cytokines, such as IL-10 and interleukin-1 receptor antagonist (IL-1ra), in decompensated liver cirrhosis and ACLF patients is significantly increased. In the late stage of ACLF, sepsis-like immune paralysis with circulating monocyte failure occurs as the main manifestation, leading to increased susceptibility to bacterial infections and resultant mortality [13, 14].

Therefore, immune regulation and promotion of liver cell regeneration are two important aspects of ACLF therapy. According to ACLF pathogenesis, early immunosuppressive therapy can block SIRS and reduce hepatocyte necrosis and extrahepatic organ dysfunction caused by immune damage. A meta-analysis indicated that early treatment of HBV-ACLF with glucocorticoids can significantly improve the survival rate without increasing the incidence of secondary infection and bleeding [15]. However, the use of hormones in ACLF patients has always been controversial. It is difficult to control the timing, dosage, and treatment period of hormone application, which may easily cause complications, such as coinfection and bleeding. On the other hand, the regeneration of hepatocytes can repair damaged/necrotic liver tissues in the absence of effective drugs. Hepatocyte growth factor (HGF) can promote the differentiation of precursor cells into liver-like cells in vitro, but its efficacy in ACLF patients is limited, which may be related to the inhibition of hepatocyte regeneration in ACLF patients [16]. Recently, Xiang et al. [17] found that imbalance of the interferon-γ (IFN-γ)/Stat1 and IL-6/Stat3 pathways is the cause of severe damage to liver regeneration in ACLF mice, and IL-22Fc can reverse the imbalance of Stat1/Stat3. IL-22Fc treatment is safe for patients with moderate and severe alcoholic hepatitis and can improve the model for end-stage liver disease (MELD) score [18]. Granulocyte colony-stimulating factor can improve the survival rate of ACLF patients by mobilizing CD34^+^ cells to promote hepatocyte regeneration, but its ability to repair liver tissue is limited [19].

Mesenchymal stem cells (MSCs) have the potential for self-renewal and multipotent differentiation. In theory, they can repair diseased cells or reconstruct normal liver cells and tissues through transplantation, providing new ideas for the treatment of liver injury diseases [20, 21]. Human umbilical cord-derived mesenchymal stem cells (hUC-MSCs) have been widely used in the clinic due to their rich sources, high proliferation potential, and low immunogenicity [22]. Increasingly, studies have confirmed that hUC-MSCs have significant positive effects on liver injury diseases, such as liver fibrosis and acute liver failure. hUC-MSCs can repair damaged liver tissues by promoting hepatocyte regeneration and inhibiting hepatocyte apoptosis [23–26]. Studies have found that hUC-MSCs can significantly improve the liver function and survival rate of HBV-ACLF patients [27, 28], while some studies have found that hUC-MSC treatment does not significantly improve the short-term prognosis of HBV-ACLF patients [29]. At present, the efficacy and mechanism of hUC-MSCs in ACLF are not clear, and related basic studies are lacking.

In the present study, ACLI and ACLF were induced in rats, and different doses of hUC-MSCs were transplanted to investigate the effectiveness and mechanism of hUC-MSC transplantation.

## Methods

### Experimental animals

Wistar rats weighing 120–150 g were obtained from the Experimental Animal Research Center of Hubei Province (Wuhan, China). Animal care and all experimental procedures were approved by ethical review by the Laboratory Animal Management and Use Committee of the Center for Disease Control of Hubei Province.

### Preparation and flow cytometry phenotyping of hUC-MSCs

hUC-MSCs were provided by Shenzhen Beike Cell Engineering Research Institute. hUC-MSCs from Wharton’s jelly were isolated by a non-enzymatic method and culture-expanded as described in previous report[30]. The infused hUC-MSCs were harvested at passage 4 and stained with trypan blue to evaluate the vitality using an automated cell counter. The vitality of final infused hUC-MSCs was ≥90%. The phenotypes of hUC-MSCs were analyzed by cytometry (Figure S1). hUC-MSCs were stained with antibodies specific for MSCs markers CD105 (BD, USA, 562408), CD90 (BD, USA, 555595), and CD73 (BD, USA, 550257) and hematopoietic cell markers CD45 (BD, USA, 555482) and CD34 (BD, USA, 550619), respectively.

### Establishment of ACLI and ACLF models and hUC-MSC transplantation

#### ACLI model

**Fig. 1:**
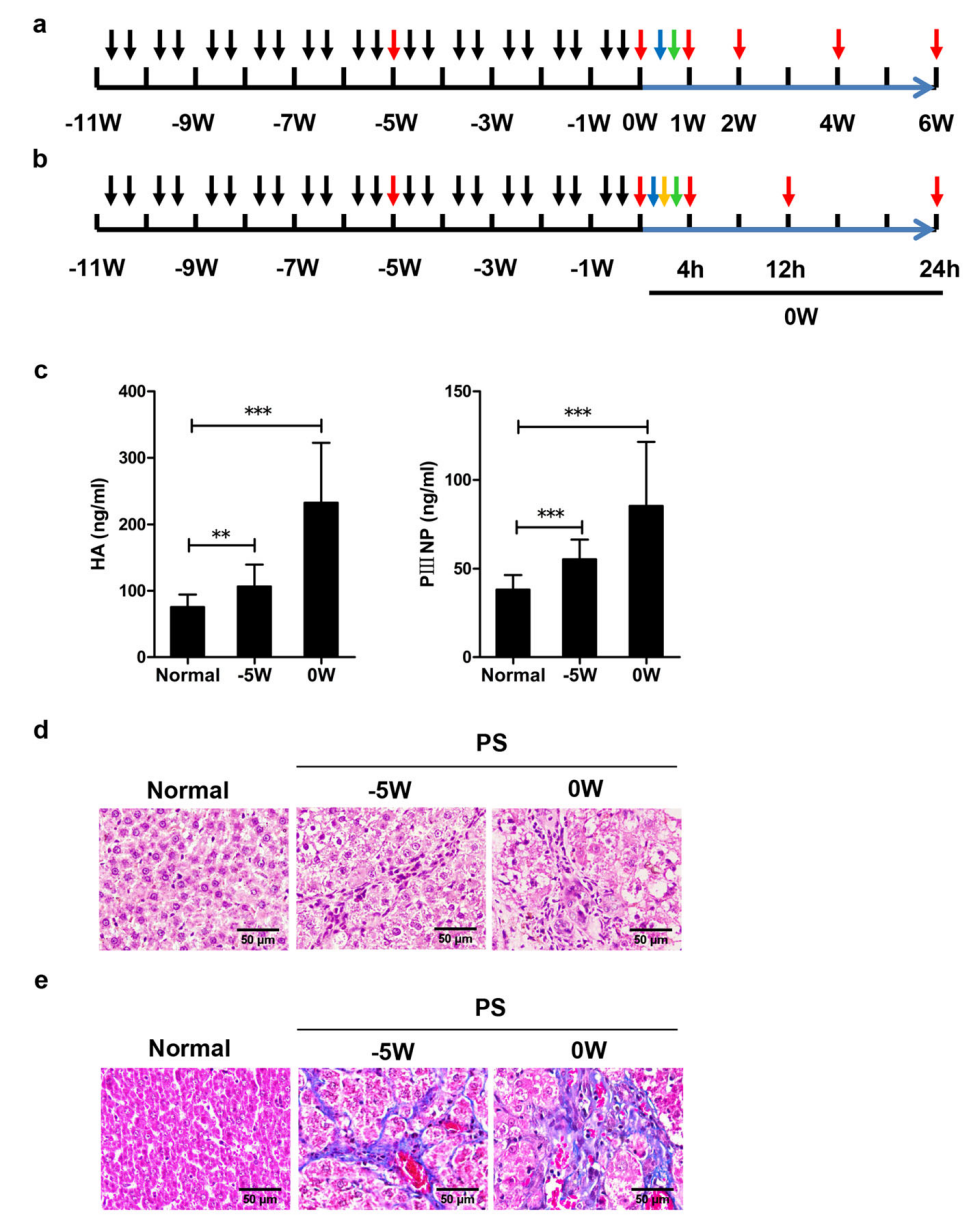
Schedules of the experiments in rats. **a** Schematic design of hUC-MSC treatment for rats with ACLI. **b** Schematic design of hUC-MSC treatment for rats with ACLF. **c–e** Liver fibrosis/cirrhosis was verified by ELISA for serum HA and PIIINP (**c**), HE staining (**d**), and Masson staining (**e**) of liver specimens from rats that received PS treatment.  PS injection.  Sacrifice.  LPS infusion.  D-GalN injection.  hUC-MSCs infusion

Rats were intraperitoneally administered porcine serum (PS) (Solarbio, China, S9060) at a dose of 0.5 mL twice per week for 11 weeks to generate an immune liver fibrosis model. After 11 weeks, rats with immune liver fibrosis were intravenously injected with lipopolysaccharide (LPS) (Sigma-Aldrich, USA, L2880) at a dose of 50 μg/kg to generate an ACLI model. After 1 h, the rats were divided into three groups. In the hUC-MSC groups, the rats underwent intravenous tail vein transplantation of hUC-MSCs at a concentration of 2×10^6^ cells/mL per rat (*n* = 12) or hUC-MSCs at a concentration of 4×10^6^ cells/mL per rat (*n* = 12). In the control group, the rats received 1 mL of 0.9% sodium chloride (*n* = 12). Three rats in each group were sacrificed at 1, 2, 4, and 6 weeks after hUC-MSCs or 0.9% sodium chloride injection, and blood samples and liver tissues were collected for biochemical and histological investigation (Fig. 1a).

#### ACLF model

Rats were intraperitoneally administered 0.5 mL PS (Solarbio, China, S9060) twice per week for 11 weeks to generate an immune liver fibrosis model. After 11 weeks, rats with immune liver fibrosis were intravenously injected with LPS at a dose of 50 μg/kg. Thirty minutes later, D-galactosamine (D-GalN) (Sigma-Aldrich, USA, G0500) was intraperitoneally injected at a dose of 600 mg/kg to induce an ACLF model [31]. After 1 h, the rats were divided into three groups. In the hUC-MSC groups, the rats underwent intravenous tail vein transplantation of hUC-MSCs at a concentration of 2×10^6^ cells/mL per rat (*n* = 20) or 4×10^6^ cells/mL per rat (*n* = 20). In the control group, control rats received 1 mL of 0.9% sodium chloride (*n* = 20). Three to four rats in each group were sacrificed at 4, 12, and 24 h after hUC-MSC injection, and blood samples and liver tissues were collected for biochemical and histological investigation (Fig. 1b).

### Determination of blood biochemical indices

The serum levels of alanine aminotransferase (ALT), aspartate aminotransferase (AST), total bilirubin (TBiL), direct bilirubin (DBiL), alkaline phosphatase (ALP), and albumin (ALB), and plasma level of ammonia were detected with an automatic analyzer (Rayto, China).

### Determination of coagulation function

The plasma prothrombin time (PT) and international normalized ratio (INR) were detected with an automatic analyzer (ACL TOP700, Spain).

### Histological examinations

Liver tissues were fixed in 4% paraformaldehyde solution. The liver tissue samples were processed using the paraffin block technique in wax, deparaffinization in xylene, and dehydration in alcohol. The samples were then sectioned (5 μm) and stained with hematoxylin-eosin (HE) to evaluate the pathological changes and Masson staining to demonstrate the collagen deposition that had occurred in the liver. The sections were then examined and photographed by light microscopy (Olympus, Japan, 200×).

### Immunohistochemistry

Immunohistochemistry staining was conducted using a previously described method [24]. The following primary antibodies were used: rabbit anti-alpha smooth muscle actin (α-SMA) mAb (1:200, Abcam, UK, ab32575), rabbit anti-desmin pAb (1:200, Abcam, UK, ab15200), rabbit anti-matrix metalloproteinase 9 (MMP9) mAb (1:1000, Abcam, UK, ab76003), rabbit anti-CD90 mAb (1:50, Abcam, UK, ab133350), rabbit anti-tissue inhibitor of metalloproteinase 1 (TIMP1) pAb (1:100, Proteintech, China, 16644-1-AP), rabbit anti-cytokeratin 18 (CK18) pAb (1:200, Proteintech, China, 10830-1-AP), rabbit anti-alpha fetoprotein (AFP) pAb (1:100, Affinity, USA, AF5134), rabbit anti-HGF pAb (1:100, Affinity, USA, DF6326), rabbit anti-proliferating cell nuclear antigen (PCNA) pAb (1:100, Affinity, USA, AF0239), rabbit anti-Phospho-Stat1 mAb (1:100, Cell Signaling Technology, USA, 8826S), and rabbit anti-Phospho-Stat3 mAb (1:100, Cell Signaling Technology, USA, 9145S). Goat anti-rabbit polyclonal antibody (1:200, Affinity, USA, S0001) was used as the secondary antibody. Three areas are randomly selected for each slice under high magnification to take pictures. ImageJ software was used to calculate the area of positive cells and the total area of the image. The area of positive staining (percent) = (area of positive cells/total area of the image) × 100%.

### Detection of fibrotic markers and cytokines

The serum levels of hyaluronic acid (HA) and N-procollagen type III peptide (PIIINP) were detected by ELISA kits (CUSABIO, China). The serum levels of TNF-α, IFN-γ, IL-6, interleukin-1β (IL-1β), transforming growth factor-β1 (TGF-β1), interleukin-4 (IL-4), IL-10, and HGF were detected by ELISA kits (RayBiotech, China).

### Quantitative real-time PCR analysis

The total RNA was isolated from liver tissues with TRIzol regent (Invitrogen, USA, 15596026) according to the manufacturer’s instructions. Approximately 1 μg of total RNA from each sample was used to synthesize cDNA using the HiScript II Q RT SuperMix for qPCR (Vazyme, China, R223-01) according to the manufacturer’s specification. Then, quantitative real-time PCR was performed by using SYBR Green Master Mix (Vazyme, China, Q111-02), with 15 s at 95°C and 60 s at 60°C for 40 cycles. GAPDH was used as the reference gene for calculations. The 2^-△△Ct^ method was used to analyze the real-time PCR data. The primers were provided in Table S1.

### Western blotting

Protein was extracted from liver tissues with RIPA lysis buffer (beyotime, China, P0013B) containing protease inhibitor (Roche, Germany, 4693116001) and phosphatase inhibitor (beyotime, China, S1873). Equal protein extracts were separated by sodium dodecyl sulfate polyacrylamide gel electrophoresis and then transferred onto polyvinylidene fluoride membranes. The membranes were incubated sequentially with appropriate primary antibodies and secondary antibodies. Immune complexes were visualized by ECL detection reagent (Applygen, China, P1050), and band intensities were determined using the BandScan software. Primary antibodies used in this study were as follows: rabbit anti-GAPDH mAb (1:1000, Cell Signaling Technology, USA, 5174S), mouse anti-Stat3 mAb (1:1000, Cell Signaling Technology, USA, 9139S), rabbit anti-Phospho-Stat3 mAb (1:2000, Cell Signaling Technology, USA, 9145S), rabbit anti-Stat1 mAb (1:1000, Cell Signaling Technology, USA, 14994S), rabbit anti-Phospho-Stat1 mAb (1:1000, Cell Signaling Technology, USA, 8826S), rabbit anti-CyclinD1 mAb (1:1000, Cell Signaling Technology, USA, 55506S), rabbit anti-c-Myc mAb (1:1000, Cell Signaling Technology, USA, 18583S), and mouse anti-Bcl2 mAb (1:200, Santa cruz, USA, sc-7382). Secondary antibodies used in this study were horseradish peroxidase-conjugated anti-mouse IgG (1:10000, Boster, China, BA1051) and anti-rabbit IgG (1:10000, Boster, China, BA1054).

### Statistical analysis

All data are expressed as the mean ± standard deviation (SD). Comparisons between two groups were assessed by the Mann-Whitney U test. Kruskal-Wallis test with Dunn’s multiple comparisons post-test analysis was used to compare among three groups. *P* <0.05 was considered statistically significant.

## Results

### hUC-MSC transplantation improved liver function in ACLI and ACLF rats

**Fig. 2:**
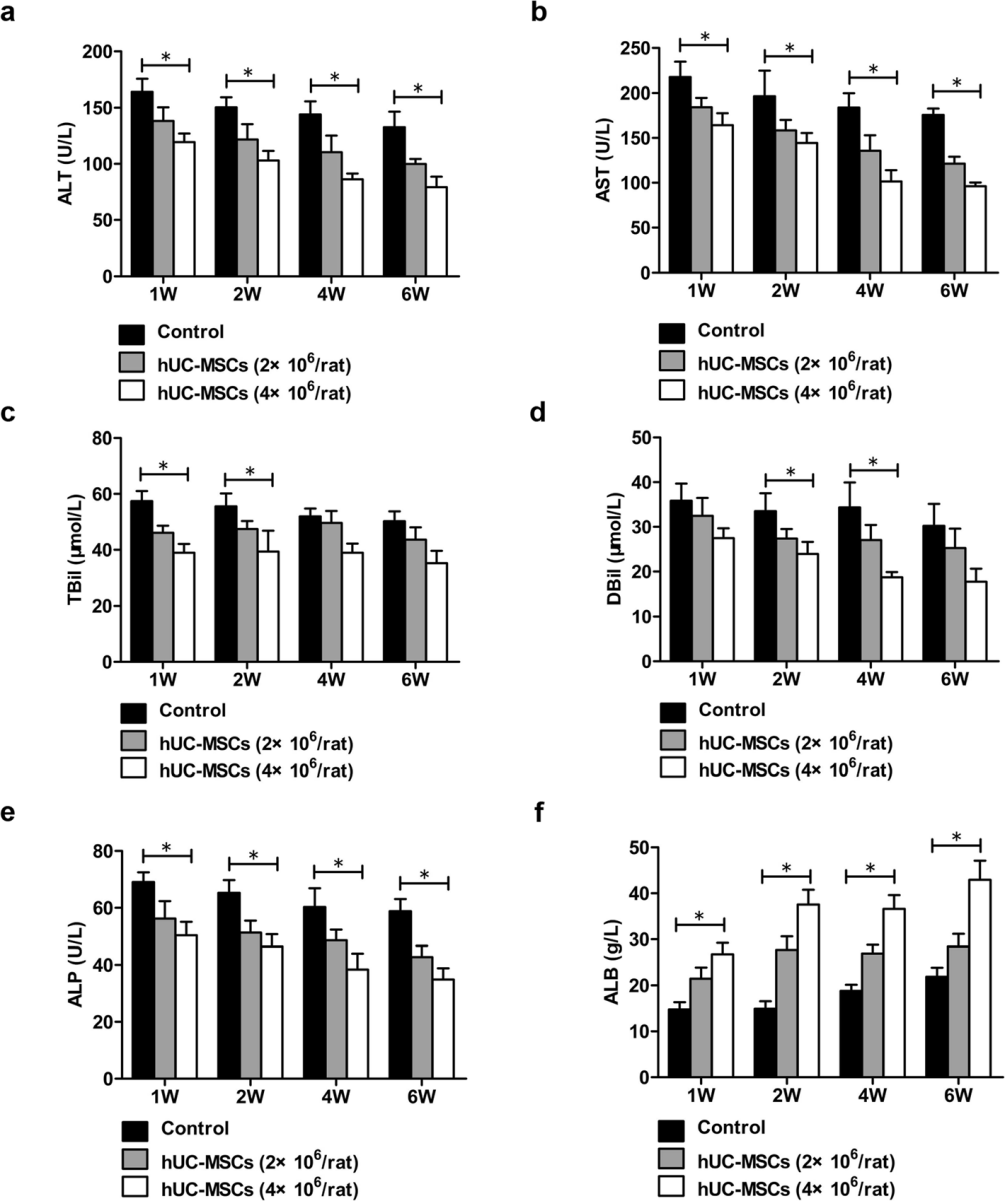
The effect of hUC-MSC transplantation in improving liver function in rats with ACLI. ACLI rats were transplanted with hUC-MSCs or 0.9% sodium chloride as controls, and serum levels of ALT (**a**), AST (**b**), TBil (**c**), DBil (**d**), ALP (**e**), and ALB (**f**) were detected with an automatic biochemical analyzer (*n* = 3/group). Data are presented as the mean ± SD. ^*^P < 0.05

At 11 weeks post-PS injection, the HA (232.1±90.39) and PIIINP (85.26±36.23) levels in the treated group were significantly increased compared to those in the normal group (Fig. 1c). In the PS-treated group, inflammatory cell infiltration was observed in the liver tissue (Fig. 1d), and Masson staining showed blue strip-like thick fibers that separated the liver lobules to form pseudolobules (Fig. 1e). These results suggested the successful establishment of rat liver fibrosis/cirrhosis models. After intravenous injection of LPS in rats with liver fibrosis, serum levels of ALT, AST, and TBil were significantly increased, while the level of ALB decreased significantly, and a large amount of inflammatory cell infiltration was observed in the portal area (Figs. 2 and 4a). These serological and histological changes lasted up to 6 weeks after LPS injection, in line with the clinicopathological characteristics of ACLI.

In ACLI rats, compared with those of control rats, the serum levels of ALT, AST, TBil, DBil, and ALP of rats receiving hUC-MSCs gradually decreased, and the ALB level gradually increased (Fig. 2a–f). After the second week, the serum ALB level in rats receiving 4×10^6^ hUC-MSCs returned to normal levels, but serum ALT, AST, and TBil levels and other indicators did not return to normal levels after hUC-MSC treatment for 6 weeks (Fig. 2a–f). While there were none of the rats died in both control group and hUC-MSC groups.

**Fig. 3:**
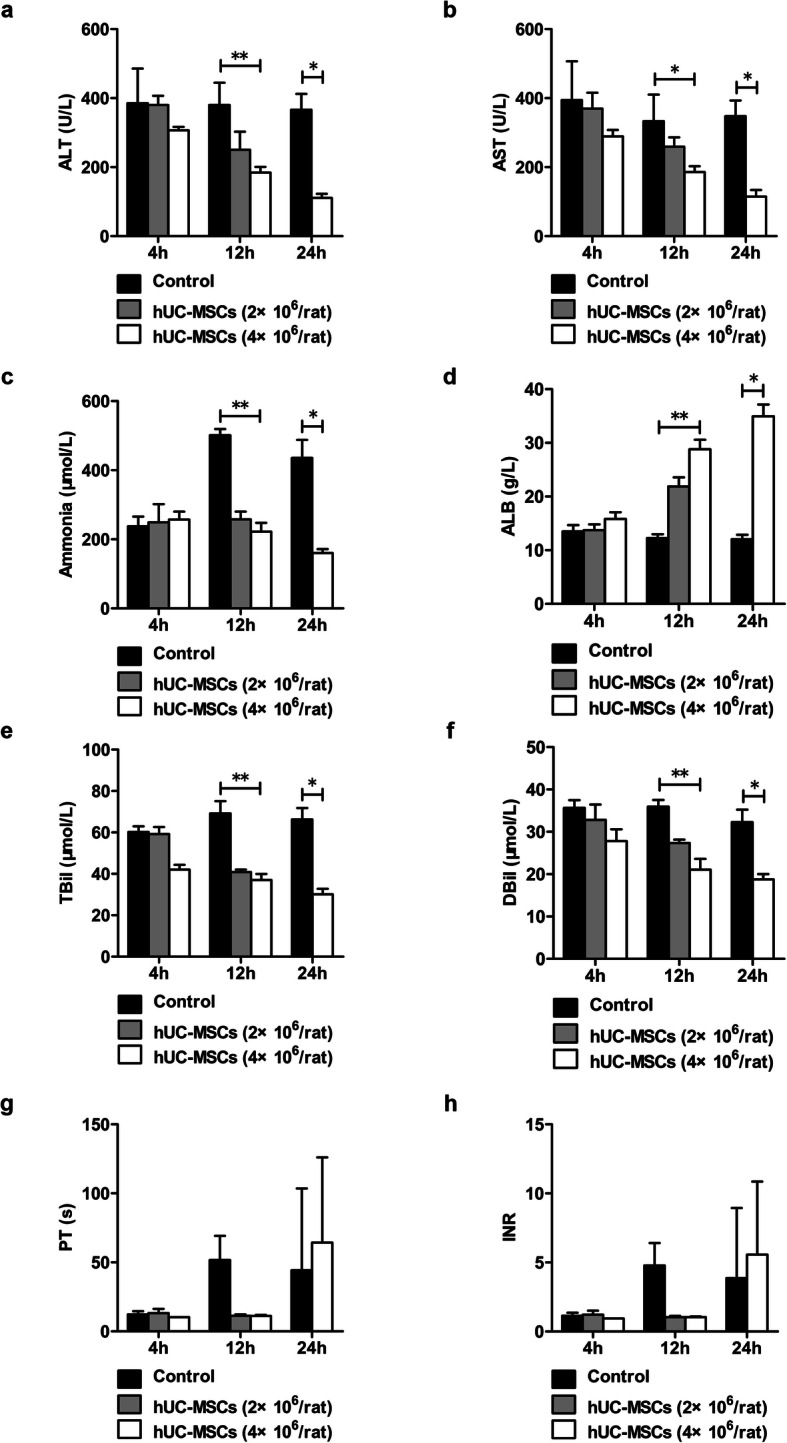
The effects of hUC-MSC transplantation on improving liver function and coagulation function in ACLF rats. ACLF rats were transplanted with hUC-MSCs or 0.9% sodium chloride as a control. Serum levels of ALT (**a**) and AST (**b**), plasma levels of ammonia (**c**), and serum levels of ALB (**d**), TBil (**e**), and DBil (**f**) were detected with an automatic biochemical analyzer, and plasma PT (**g**) and INR (**h**) were detected with a coagulation analyzer (4h, *n* = 3/group; 12h and 24h, *n* = 4/group). Data are presented as the mean ± SD. ^*^P < 0.05, ^**^P < 0.01

Four hours after LPS and D-GalN injection in rats with liver fibrosis, rat serum ALT, and AST levels reached 400 U/L, and the TBil level reached 60 μmol/L, with increased plasma levels of ammonia and decreased levels of ALB. PT was significantly prolonged to more than 50 s, and INR increased to more than 5.0 (Fig. 3). Histological examination results showed a large amount of inflammatory cell infiltration and hepatocyte patchy necrosis in liver tissue (Fig. 5). The activity of the rats was significantly reduced. The animal status was extremely poor, with a large number of short-term deaths. These results were in line with the clinical and pathological characteristics of ACLF. After treatment with hUC-MSCs for 12 h, the levels of ALT, AST, TBil, and ammonia in ACLF rats decreased significantly, and the level of ALB increased significantly. After treatment with hUC-MSCs for 24 h, the levels of ALT, AST, TBil, and ammonia of rats receiving 4×10^6^ hUC-MSCs were significantly lower than those of rats receiving 2×10^6^ hUC-MSCs (Fig. 3a–e). Interestingly, the serum level of ALB in rats receiving 4×10^6^ hUC-MSCs returned to near normal levels within 24 h (Fig. 3d). Moreover, after 12 h of hUC-MSC treatment, the PT and INR of rats in the hUC-MSC group dropped to normal levels. However, due to the small sample sizes and large standard deviations, there were no statistical differences in PT and INR values among control group and hUC-MSC groups. 24 hours later, the PT and INR values of the surviving rats could not be measured by statistics due to large disparities (Fig. 3g, h). Nine rats in the control group, thirteen rats in 2×10^6^ hUC-MSC group, and nine rats in 4×10^6^ hUC-MSC group died before sacrifice.

When stained with antibody specific for human CD90 (a marker of MSC), a large amount of CD90-positive cells were presented in the liver tissues from both ACLI and ACLF rats received hUC-MSC therapy, while not in the liver tissues from rats received 0.9% sodium chloride (Figure S2). This result revealed that the intravenously transplanted hUC-MSCs did integrate into the injured liver in both ACLI and ACLF rats.

### hUC-MSC transplantation protected against hepatic injury

**Fig. 4:**
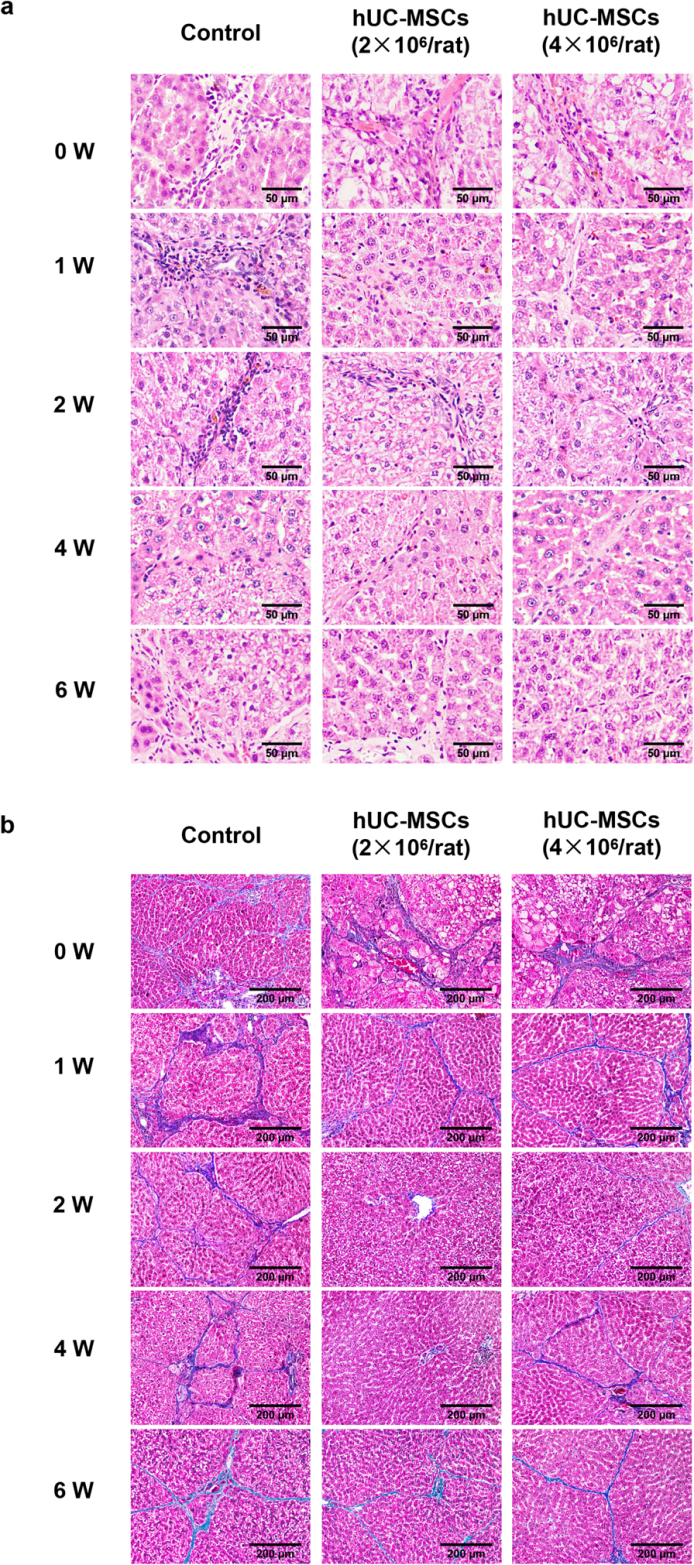
Histopathological recovery of liver tissues from rats with ACLI transplanted with hUC-MSCs. ACLI rats were transplanted with hUC-MSCs or 0.9% sodium chloride as a control, and liver sections were used for histological investigation with HE staining (**a**) or Masson staining (**b**) and microscopic examination (*n* = 3/group). Representative photographs are shown for the liver histological presentations of rats with ACLI transplanted with hUC-MSCs or 0.9% sodium chloride as a control at weeks 0, 1, 2, 4, and 6

The results of HE staining showed that in ACLI rats at 0 week, a large number of inflammatory cells infiltrated the liver in the three groups of rats, and vacuolar degeneration and disorderly arrangement were observed in hepatocytes. Compared with control rats, ACLI rats had fewer inflammatory cells and degenerated cells in the liver at 1 week after hUC-MSC injection (Fig. 4a). The results of Masson staining showed that collagen deposition in the livers of rats injected with hUC-MSCs gradually decreased. Among them, at 2 and 4 weeks, there was no obvious collagen deposition in the liver tissue of the 2×10^6^ hUC-MSC treatment group rats, while collagen deposition in the 4×10^6^ hUC-MSC treatment group rats was not completely relieved (Fig. 4b).

**Fig. 5:**
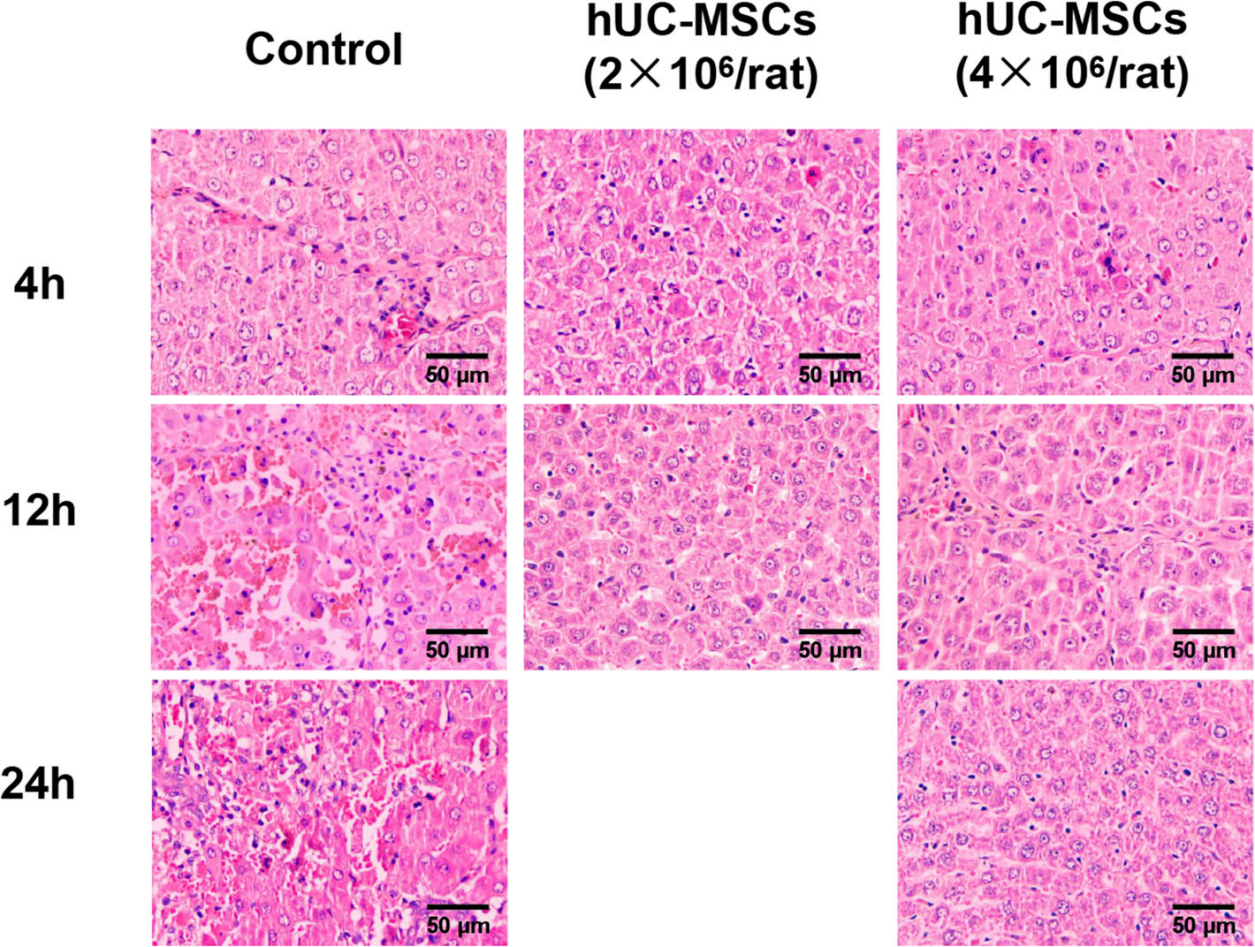
Histopathological recovery of liver tissues from ACLF rats transplanted with hUC-MSCs. ACLF rats were transplanted with hUC-MSCs or 0.9% sodium chloride as a control, and liver sections were used for histological investigation with HE staining and microscopic examination (4h, *n* = 3/group; 12h and 24h, *n* = 4/group). Representative photographs are shown for the liver histological presentations of ACLF rats transplanted with hUC-MSCs or 0.9% sodium chloride as a control at hours 4, 12, and 24

A large number of infiltrating inflammatory cells and necrotic hepatocytes appeared in the liver of ACLF rats, and the number of inflammatory cells gradually increased from 4 to 24 h. The degree of liver tissue necrosis was significantly enhanced, while the rats injected with hUC-MSCs had fewer inflammatory infiltrating cells and necrotic liver cells (Fig. 5).

### hUC-MSC transplantation inhibited the production of extracellular matrix (ECM) in the liver of ACLI rats

**Fig. 6:**
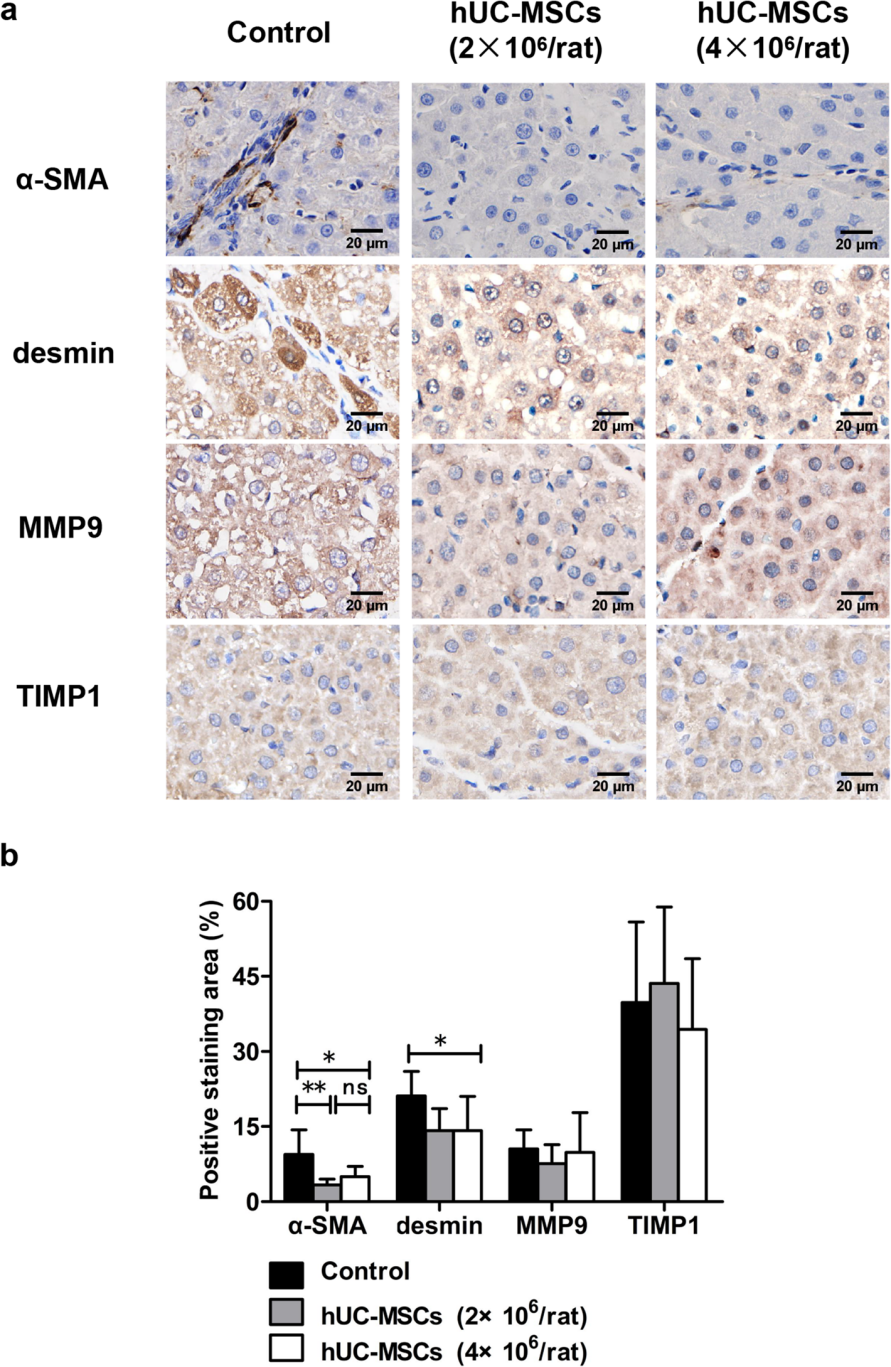
The effects of hUC-MSC transplantation on liver fibrosis in rats with ACLI. ACLI rats were transplanted with hUC-MSCs or 0.9% sodium chloride as a control, and liver sections from ACLI rats 6 weeks post-hUC-MSC transplantation were used for immunohistochemical staining of α-SMA, desmin, MMP9, and TIMP1 and microscopic examination (*n* = 3/group). Representative photographs are shown (**a**). Positive staining was quantified and is presented as the mean ± SD (**b**). ^*^P < 0.05, ^**^P < 0.01

The immunohistochemistry results showed that α-SMA and desmin expression was increased in the liver tissue of ACLI rats, while after treatment with hUC-MSCs, the α-SMA and desmin expression was significantly reduced (*P* < 0.05). There was no significant difference between 2×10^6^ hUC-MSCs and 4×10^6^ hUC-MSCs in inhibiting α-SMA and desmin expression in rat liver. However, the expression levels of MMP9 and TIMP1 in rat livers were not significantly different between the control group and the hUC-MSC treatment group (Fig. 6).

### hUC-MSC transplantation promoted regeneration of hepatocytes in ACLF rats

**Fig. 7:**
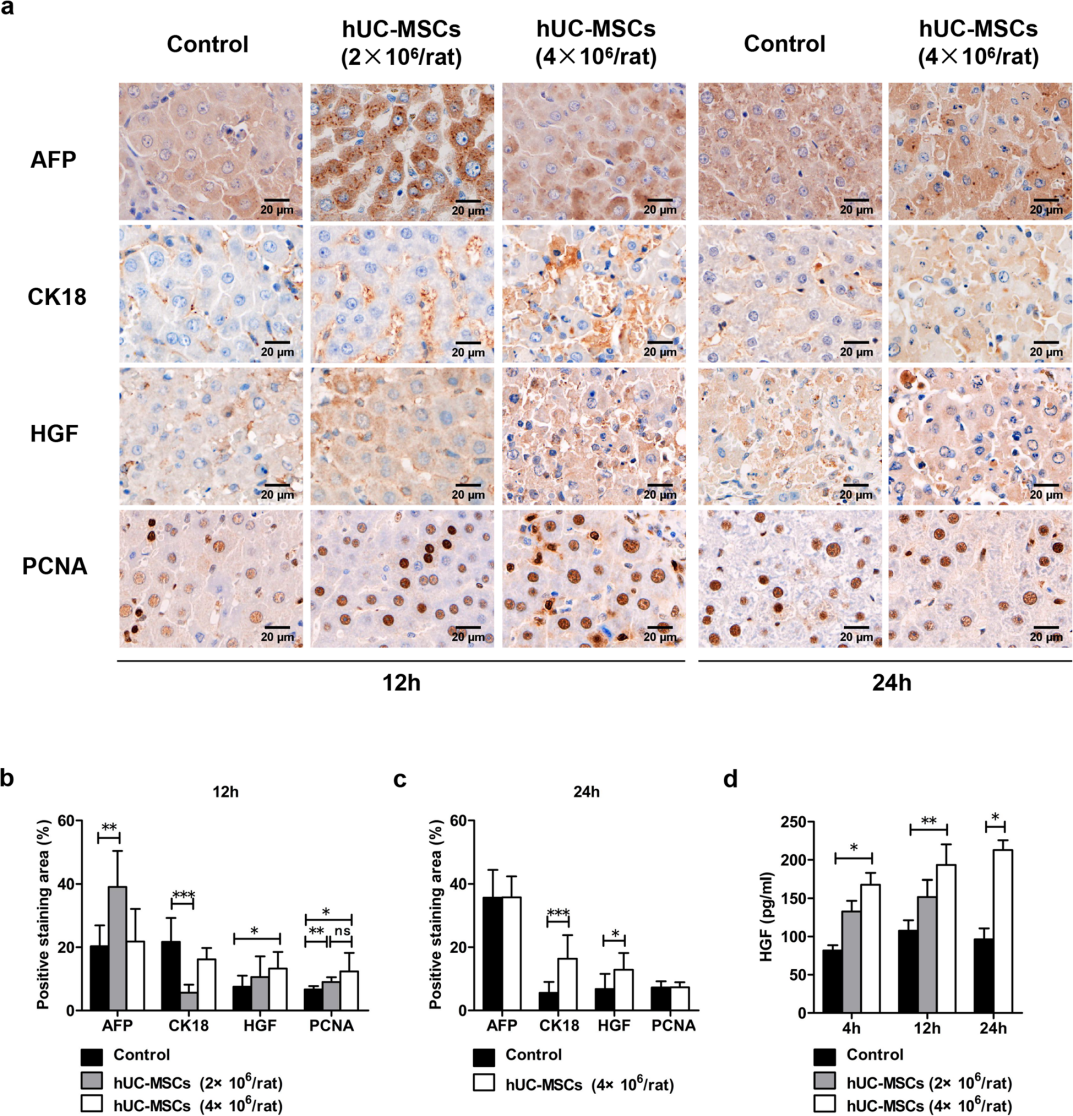
The effects of hUC-MSC transplantation on hepatocyte regeneration in ACLF rats. Liver sections from ACLF rats 12 and 24 h post-hUC-MSC transplantation or 0.9% sodium chloride injection as a control were used for immunohistochemical staining of AFP, CK18, HGF, and PCNA and microscopic examination (*n* = 4/group). Representative photographs are shown (**a**). Positive staining was quantified and is presented as the mean ± SD (**b, c**). **d** Serum levels of HGF were detected by ELISA (4h, *n* = 3/group; 12h and 24h, *n* = 4/group). Data are presented as the mean ± SD. ^*^P < 0.05, ^**^P < 0.01, ^***^P < 0.001

Twelve hours after treatment with hUC-MSCs or 0.9% sodium chloride, the expression levels of AFP, HGF, and PCNA in the hUC-MSC treatment group were significantly increased compared with those in rat liver tissues in the control group (*P* < 0.05). The expression of HGF and PCNA in rats receiving 4×10^6^ hUC-MSCs or 2×10^6^ hUC-MSCs showed no significant difference, but strangely, there was no significant increase in the expression of AFP in the 4×10^6^ hUC-MSC treatment group at 24 h. At 24 h after hUC-MSC transplantation, the expression of AFP and PCNA in liver tissues of the control group and in those of the 4×10^6^ hUC-MSC treatment group was not significantly different, while the expression of CK18 and HGF in liver tissues of the rats treated with 4×10^6^ hUC-MSCs was significantly higher than that in in liver tissues of the rats in the control group (*P* < 0.05) (Fig. 7a–c). In addition, the serum level of HGF in rats treated with hUC-MSCs (especially 4×10^6^ hUC-MSCs) was significantly higher than that in the control rats from 4 to 24 h (*P* < 0.001) (Fig. 7d).

### hUC-MSC transplantation inhibited the production of proinflammatory cytokines and promoted the production of antiinflammatory cytokines

**Fig. 8:**
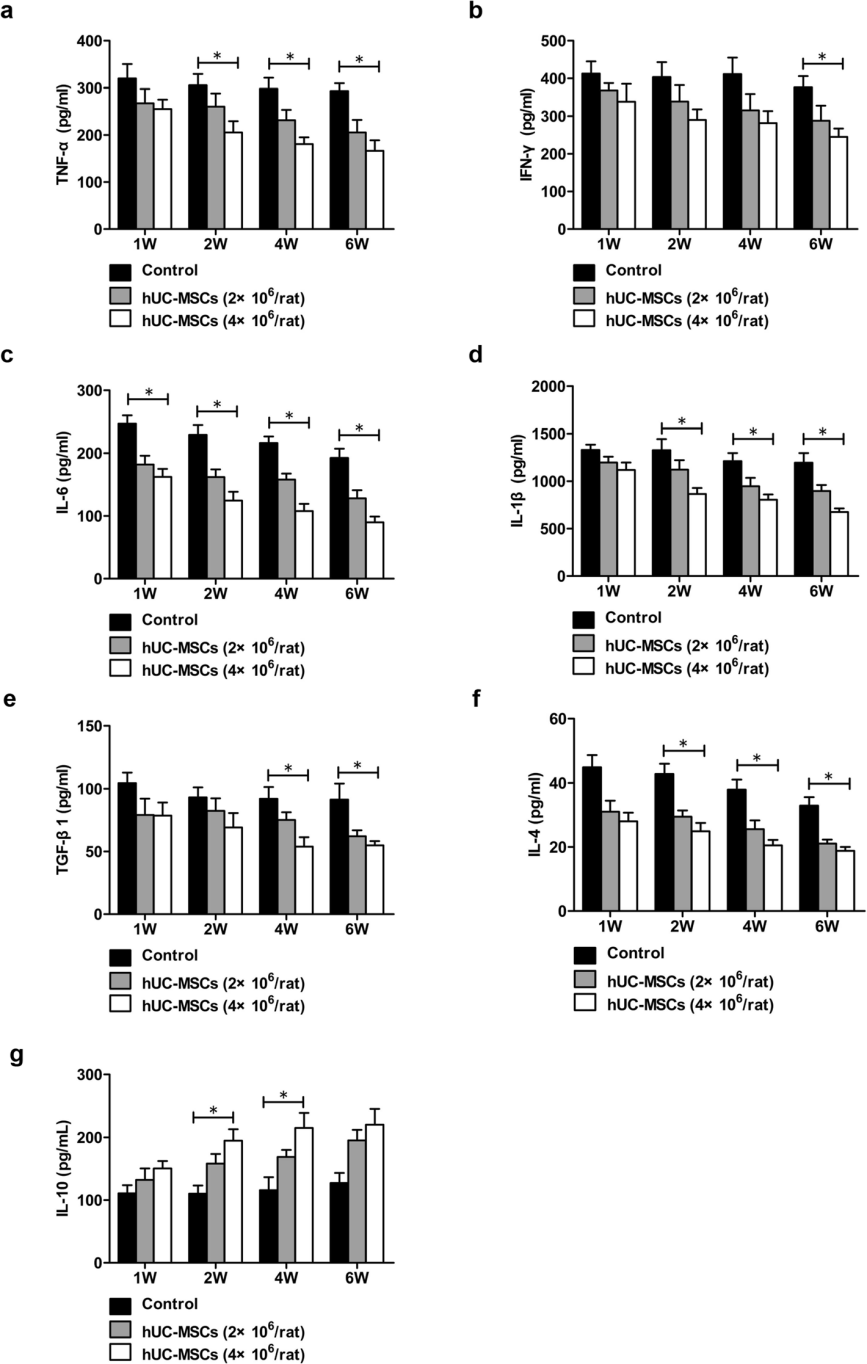
The effects of hUC-MSC transplantation on the production of proinflammatory cytokines and antiinflammatory cytokines in rats with ACLI. ACLI rats were transplanted with hUC-MSCs or 0.9% sodium chloride as a control. The serum levels of the cytokines TNF-α (**a**), IFN-γ (**b**), IL-6 (**c**), IL-1β (**d**), TGF-β1 (**e**), IL-4 (**f**), and IL-10 (**g**) were detected by ELISA at weeks 1, 2, 4, and 6 (*n* = 3/group). Data are presented as the mean ± SD. ^*^P < 0.05

After injection with hUC-MSCs in ACLI rats, serum levels of TNF-α, IFN-γ, IL-6, IL-1β, TGF-β1, and IL-4 gradually decreased, while IL-10 levels gradually increased (Fig. 8).

**Fig. 9:**
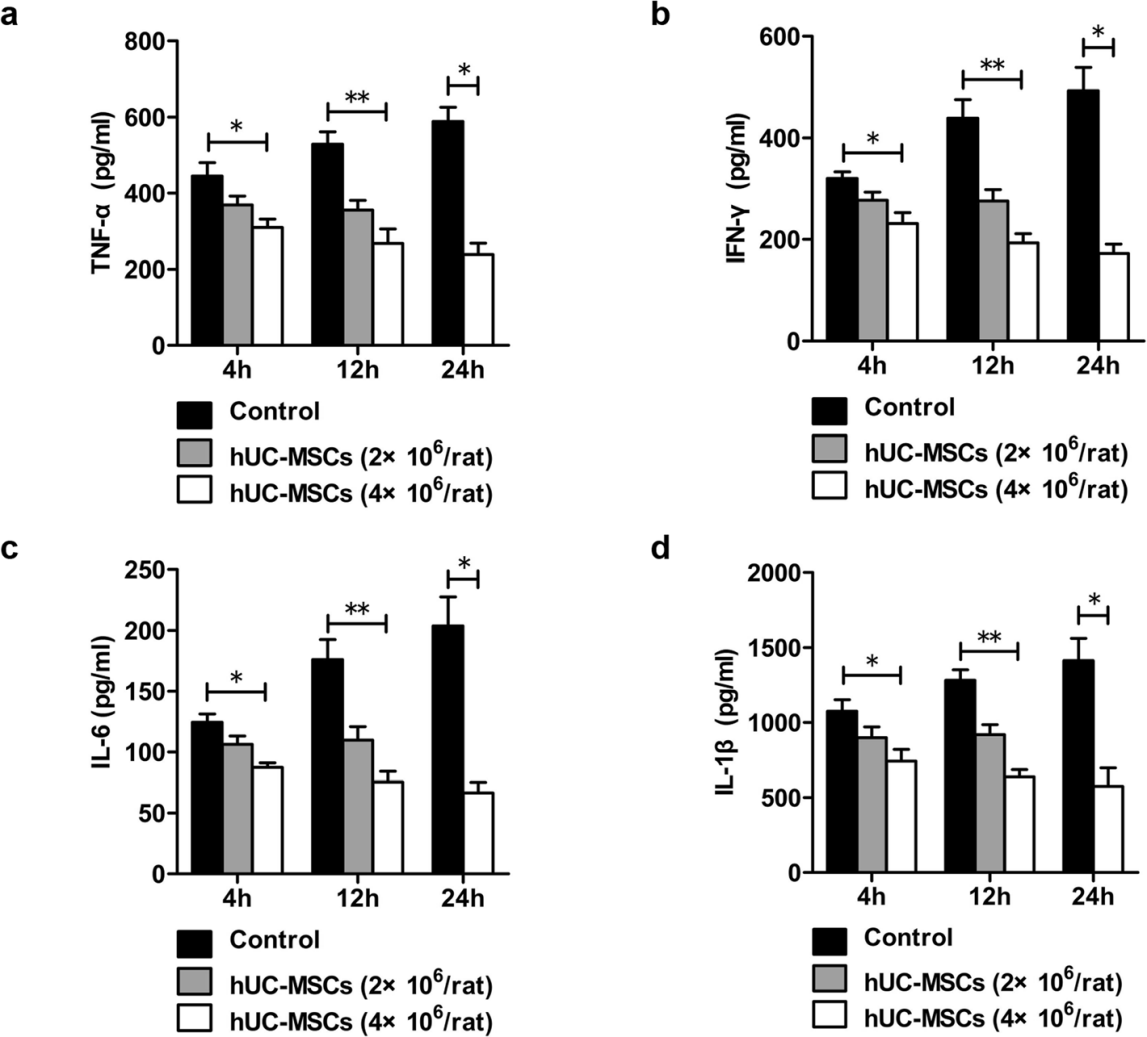
The effects of hUC-MSC transplantation on the production of proinflammatory cytokines in ACLF rats. ACLF rats were transplanted with hUC-MSCs or 0.9% sodium chloride as a control. The serum levels of the cytokines TNF-α (**a**), IFN-γ (**b**), IL-6 (**c**), and IL-1β (**d**) were detected by ELISA at hours 4, 12, and 24 (4h, *n* = 3/group; 12h and 24h, *n* = 4/group). Data are presented as the mean ± SD. ^*^P < 0.05, ^**^P < 0.01

The serum levels of TNF-α, IFN-γ, IL-6, and IL-1β in ACLF rats showed gradual increases from 4 to 24 h. Four hours after treatment with hUC-MSCs, the serum levels of TNF-α, IFN-γ, IL-6, and IL-1β in ACLF rats decreased significantly and continued to decrease over time. After 12 h, the serum levels of TNF-α, IFN-γ, IL-6, and IL-1β in rats treated with 4×10^6^ hUC-MSCs were lower than those in rats treated with 2×10^6^ hUC-MSCs although there was no statistically different (Fig. 9).

### hUC-MSC transplantation downregulated Notch and Stat1/Stat3 signaling in ACLF rats

**Fig. 10:**
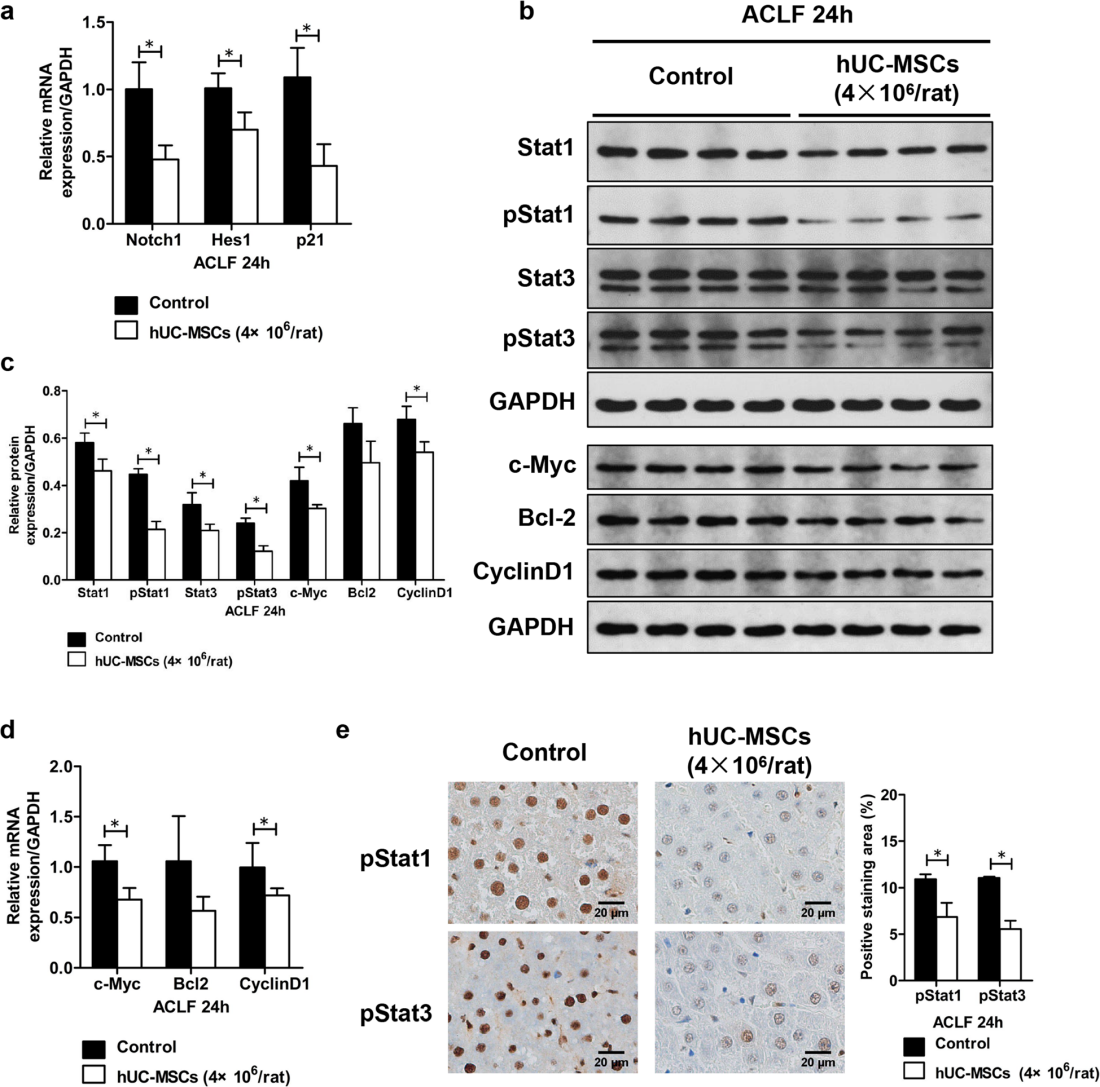
The effects of hUC-MSC transplantation on Notch signaling pathway-related genes and Stat1/Stat3 signaling molecules in ACLF rats. Total RNA and protein were extracted from the liver tissues from rats with ACLF transplanted with hUC-MSCs or 0.9% sodium chloride as a control(*n* = 4/group). The mRNA expression levels of Notch1, Hes1, and P21 were detected by quantitative PCR (**a**). Total protein was subjected to western blotting analysis. Relative protein expression of Stat1, pStat1, Stat3, pStat3, c-Myc, Bcl2, and CyclinD1 were quantitated (**b**, **c**). The mRNA expression levels of c-Myc, Bcl2, and CyclinD1 were detected by quantitative PCR (**d**). Liver sections were used for immunohistochemically staining of pStat1 and pStat3. Representative photographs and the quantitative analysis were shown (**e**). Data were presented as the mean ± SD. *P < 0.05

In ACLF rats, compared with the rats received 0.9% sodium chloride, significantly decreased mRNA levels of Notch1, Hes1, and p21 were evidenced in liver tissues from rats received 4×10^6^ hUC-MSCs 24 h post-transplantation (*P* < 0.05) (Fig. 10a). In ACLI rats, the mRNA levels of Notch1, Hes1, and p21 were all lower in liver tissues from rats received 4×10^6^ hUC-MSCs than in rat liver tissues from rats received 0.9% sodium chloride 24 h and 1-week post-transplantation, although there was no statistically different (Figure S3a).

Results from western blotting showed significantly decreased protein levels of Stat1, pStat1, Stat3, pStat3, c-Myc, and CyclinD1 in liver tissues from rats received 4×10^6^ hUC-MSCs 24 h post-transplantation, when compared with those in liver tissues from rats in the control group (*P* < 0.05) (Fig. 10b, c). qPCR also demonstrated significantly decreased mRNA levels of c-Myc and CyclinD1 in liver tissues from rats received 4×10^6^ hUC-MSCs, compared with those in rat liver tissues in the control group (*P* < 0.05) (Fig. 10d). Furthermore, immunohistochemistry results showed that the levels of pStat1 and pStat3 were significantly decreased in liver tissues from rats received 4×10^6^ hUC-MSCs, compared with those from rats in the control group (*P* < 0.05) (Fig. 10e). The protein levels of Stat1, pStat1, Stat3, and pStat3 were lower in liver tissues from rats received 4×10^6^ hUC-MSCs than those from rats received 0.9% sodium chloride 4 h post-transplantation, although there was no statistically different (Figure S3b). In ACLI rats, the protein levels of Stat1, pStat1, Stat3, pStat3, c-Myc, Bcl2, and CyclinD1 were all lower in liver tissues from rats received 4×10^6^ hUC-MSCs than in rat liver tissues from rats received 0.9% sodium chloride, although there was no statistically different (Figure S3c-f).

## Discussion

This study used ACLI and ACLF rat models based on liver fibrosis and showed that hUC-MSCs can significantly improve liver function, coagulation function, and the degree of liver damage. hUC-MSCs can also promote ALB synthesis in the liver, necrotic tissue repair, collagen fiber degradation, and liver cell regeneration. They inhibit the production of ECM and inflammatory cytokines and promote the production of antiinflammatory cytokines. These findings confirmed that hUC-MSCs have potential therapeutic effects on promoting liver regeneration in rats with ACLI or ACLF, mediated most likely by inhibiting Notch signaling and Stat1/Stat3 pathway.

ACLF occurs mostly in CLD based on liver fibrosis/cirrhosis. Under the effects of acute injury due to alcohol consumption, hepatotropic virus infection or drugs, continuous inflammation, SIRS, and cytokine storms occur, which play a central role in the pathogenesis of liver failure and subsequent organ failure [3]. The ACLF rat model was induced by LPS/D-GalN injection following liver fibrosis/cirrhosis induced by PS administration, which can effectively simulate human ACLF [31]. In the present study, after 11 weeks of PS injection, a large number of collagen fibers were deposited in the liver tissue of rats, and pseudolobules were formed, accompanied by obvious inflammatory cell infiltration and increased serum levels of HA and PIIINP, confirming the development of liver fibrosis/early liver cirrhosis. After injection with LPS/D-GalN, rat serum levels of ALT, AST, and TBil and plasma ammonia levels increased significantly, plasma PT was obviously prolonged, and a large number of infiltrating inflammatory cells and necrotic hepatocytes were observed in liver tissues. These results were similar to the clinical and pathological changes in ACLF, indicating that the ACLF rat model was successfully constructed. On the other hand, in addition to ACLF, ACLI that occurs on the basis of CLD is more commonly clinically manifested as chronic hepatitis activity or active cirrhosis with liver cell inflammation and a small amount of necrosis [32]. In this study, in rats with liver cirrhosis, only LPS injected without D-GalN resulted in a moderate increase in ALT, AST, and TBil levels, accompanied by a decrease in ALB levels, without changes in coagulation function, blood ammonia content, or hepatocyte necrosis, suggesting the successful establishment of a non-ACLF (ACLI) rat model.

ALT and AST are soluble enzymes in hepatocytes. When the hepatocyte membrane is damaged, the permeability of the cell membrane increases, causing the release of intracellular ALT and AST into the blood. Therefore, the serum levels of ALT and AST are the most direct indicators reflecting the degree of hepatocyte damage. Zhang et al. [24] found that after hUC-MSC treatment in a rat model of acute liver failure (ALF), the serum levels of ALT, AST, and TBil decreased significantly. The present study found that in ACLI rats treated with hUC-MSCs, the serum levels of ALT and AST gradually decreased within 1–6 weeks and that other serological indicators of liver function, such as TBil, DBil, and ALP, also gradually decreased. Furthermore, after treatment with hUC-MSCs, the serum levels of ALT and AST in ACLF rats decreased more rapidly. These results are consistent with the effects of hUC-MSC treatment in ALF rats. High serum levels of bilirubin and coagulation dysfunction/failure are the most prominent clinical features of ACLF [2]. After treatment with hUC-MSCs in ACLF rats, a rapid decline in TBil levels was observed at 4 h, and coagulation function began to return to normal at 12 h, manifesting as PT and INR normalization. These results are consistent with the study of Guo et al. [33], which indicated that hUC-MSCs can promote bilirubin metabolism and liver clotting factor synthesis. In this study, the values of PT and INR in ACLF rats at 12 and 24 h after hUC-MSC treatment showed large variations. Therefore, there were no significant differences between the hUC-MSC treatment group and the control group in the values of PT and INR. Generally, the large standard deviations may cover the difference between groups. This result may be related to the small number of animals and the control of the hUC-MSC infusion process. A larger sample is needed to confirm this point.

Serum ALB is mainly synthesized by the liver. Therefore, ALB is also an important indicator reflecting the synthesis function of the liver. The serum level of ALB increased steadily after hUC-MSC treatment in ACLI rats and reached a near normal level at 6 weeks. In ACLF rats, the serum level of ALB continued to increase at 12 h after hUC-MSC treatment and returned to near normal levels at 24 h, suggesting that hUC-MSC treatment can promote the recovery of liver synthesis function, which is essential for ACLF treatment and liver repair. Studies have found that hUC-MSCs can significantly increase the short-term and long-term levels of ALB in HBV-ACLF patients [27, 28], which is consistent with the present study.

Elevated blood ammonia is an important mechanism for ACLF to develop hepatic encephalopathy. Due to liver failure, the metabolism of ammonia, thiols, short-chain fatty acids, and other products is disordered [34]. Lin et al. [35] found that the level of blood ammonia in fulminant liver failure pigs was significantly reduced after MSC treatment, suggesting that MSCs can improve the liver metabolism of ammonia. In this study, the increased level of blood ammonia in ACLF rats after hUC-MSC treatment was alleviated, suggesting that hUC-MSCs can improve the liver metabolism of ammonia and other products and may play an important role in the prevention and treatment of hepatic encephalopathy.

Zhang et al. [36] found that the degeneration, necrosis, and fatty degeneration of hepatocytes in cirrhotic rats treated with hUC-MSCs were reduced and that collagen deposition was inhibited. hUC-MSC treatment can also reduce liver inflammation and hepatocyte necrosis in ALF rats [24]. The present study showed that hUC-MSCs can reduce liver inflammatory cell infiltration and collagen deposition in ACLI and ACLF rats and that necrotic hepatocytes in ACLF rats treated with hUC-MSCs basically disappeared. This result is also consistent with the above findings, indicating that hUC-MSC treatment can alleviate pathological damage, such as hepatocyte degeneration and necrosis, and promote collagen degradation. Therefore, hUC-MSCs can repair damaged liver tissue to a certain extent.

Activated hepatic stellate cells (HSCs) secrete a large amount of ECM, which eventually leads to collagen formation and fibrosis/cirrhosis [37]. HSCs are the main cellular targets of antifibrosis therapy, and the increased α-SMA and desmin levels are considered to be signs of HSC activation [38, 39]. This study found that the α-SMA and desmin expression levels in the liver of ACLI rats after 6 weeks of hUC-MSC treatment were significantly lower than those in the liver of the control rats, indicating that hUC-MSCs may play a repair role by inhibiting the activation of HSCs. In addition, there were no significant differences in the α-SMA and desmin expression with different doses of hUC-MSCs, suggesting that hUC-MSCs have no dose-dependent inhibitory effect on HSC activation. Activated HSCs secrete matrix metalloproteases (MMPs), such as MMP2, MMP3, MMP9, and tissue inhibitors of matrix metalloproteases (TIMPs), such as TIMP1 and TIMP2. The balance of MMP/TIMP has a significant impact on ECM degradation [39, 40]. This study showed that there were no significant differences in the expression of MMP9 and TIMP1 in the liver of ACLI rats between the hUC-MSC treatment group and the control group, suggesting that hUC-MSCs may not exert antifibrotic effects by regulating the expression of these two proteins. Kupffer cells can release TGF-β1 after liver injury, and TGF-β1 can induce HSC proliferation and differentiation [41, 42]. Studies have shown that hUC-MSCs can exert antifibrosis function in a paracrine manner through the TGF-β1/Smad signaling pathway [24, 43, 44]. This study showed that serum levels of TGF-β1 in ACLI rats treated with hUC-MSCs were significantly reduced, suggesting that hUC-MSCs may inhibit HSC activation by downregulating TGF-β1 expression, thereby exerting an antifibrotic effect. Notch signaling plays critical roles in development, tissue homeostasis, and human diseases [45]. Recent advances found that the Notch pathway is involved in liver regeneration and repair, liver fibrosis, and metabolism. Inhibition of Notch signaling can significantly inhibit HSC activation and fibrosis progression [46, 47]. It has been found in zebrafish that inhibition of Notch signaling can promote the differentiation of liver progenitor cells into hepatocytes, thus promoting liver regeneration [48].In this study, hUC-MSC transplantation significantly decreased Notch1 receptor and downstream target genes Hes1 and p21 of Notch pathway in ACLF rats, suggesting that the effect of hUC-MSCs in inhibiting liver fibrosis and promoting liver regeneration may be mediated by downregulating of Notch signaling. In ACLI rats, hUC-MSC transplantation also led to a downregulated Notch signaling, the absence of significant differences when compared with those in the control group may be a result of the small number of animals.

Sublarge hepatic necrosis is the main pathological feature of ACLF and the pathological basis of liver failure [12]. The liver failure prognosis depends mainly on the regeneration ability of hepatocytes. HGF is a paracrine cell growth factor synthesized by liver nonparenchymal cells after liver injury. It is essential to promote liver regeneration and can stimulate hepatocyte regeneration to alleviate liver failure [49]. This study showed that the expression of AFP and PCNA in the liver of ACLF rats increased significantly at 12 h after hUC-MSC injection and that the hepatocyte proliferation marker CK18 also increased significantly at 24 h after hUC-MSC treatment. Furthermore, the expression of HGF in both liver tissue and serum increased significantly, suggesting that hUC-MSCs can promote liver cell regeneration, possibly by promoting HGF secretion to enhance the regeneration ability of hepatocytes in ACLF rats. MSCs can treat fulminant liver failure, and the activation of the DLL4-Notch pathway mediated by MSCs promotes the liver repair process [50]. Due to disruption of the IL-6/Stat3 pathway, excessive activation of the IFN-γ/Stat1 pathway leads to weakened liver regeneration in ACLF patients [17]. In this study, hUC-MSC treatment for ACLF rats led to significantly deceased IFN-γ in plasma and downregulated IFN-γ/Stat1 signaling in the liver, indicating that hUC-MSCs can inhibit IFN-γ/Stat1 signaling pathway. Xiang et al. found that the expression of pStat3 was decreased in ACLF mice, IL-22c-mediated pStat3 activation may lead to enhanced liver regeneration, which will be beneficial to ACLF. While pStat3 levels as presented in western blots varies among the animals in the study. While high IL-6 level is positively correlated with poor prognosis in ACLF patients [13, 51]. It is generally believed that the increase in IL-6 level reflects the active of immune response and inhibits liver regeneration [14, 52]. Similarly, in this study, ACLF rats displayed significantly activated IL-6/Stat3 signaling, and hUC-MSC transplantation mediated downregulation of IL-6/Stat3 pathway may favor the recovery of ACLF. Therefore, interrupting the excessive activated IFN-γ/Stat1 and IL-6/Stat3 pathway may contribute to the effect of hUC-MSCs in antiinflammation and promoting hepatocyte regeneration.

In this study, there were no differences in the expression of AFP and PCNA in the liver tissues of ACLF rats in the control group and in the liver tissues of rats in the high-dose hUC-MSC group after 24 h. On the one hand, the liver itself may have certain reactive hepatocyte regeneration after liver injury; on the other hand, this result may be related to the small sample size and the control of the hUC-MSC infusion process. As mentioned above, the PT and INR of ACLF rats treated with hUC-MSCs or 0.9% sodium chloride showed a large variation after 24 h, and there was no significant difference between the hUC-MSC treatment group and the control group.

SIRS is the initiating factor of ACLF and the driving factor of disease progression [11]. When affected by inflammatory factors, MSCs can migrate to inflammatory tissues and release a variety of soluble proteins, such as nitric oxide, prostaglandin E2 (PGE2), IL-6, and IL-10. These soluble proteins can promote the proliferation of a variety of immune cells and modulate their functions. PGE2 can promote the production of the antiinflammatory cytokine IL-10 in dendritic cells (DCs) and decrease the production of TNF-α and IFN-γ. In addition, PGE2 can also decrease the production of IFN-γ and IL-4 in T helper 1 and T helper 2 cells, thereby exerting immune regulation to promote tissue repair [53, 54]. The present study showed that hUC-MSC treatment significantly inhibited the production of the proinflammatory cytokines TNF-α, IFN-γ, IL-6, IL-1β, TGF-β1, and IL-4 and promoted the production of the antiinflammatory cytokine IL-10. These results further confirmed the immunomodulatory effect of hUC-MSCs in ACLF-related SIRS. This immunomodulatory effect of hUC-MSCs can block further damage to the liver and extrahepatic organs caused by SIRS, which is conducive to ACLF control.

In addition, this study showed that high-dose hUC-MSC treatment was superior to low-dose hUC-MSC treatment in improving the liver function and inhibiting the production of proinflammatory cytokines in ACLI and ACLF rats. These results indicated that the high dose may have better efficacy, especially in the treatment of ACLF, which may exert comprehensive effects, such as immune regulation, liver function improvement, antifibrosis, and hepatocyte regeneration promotion. The specific infusion dose needs to be further optimized.

## Conclusions

In summary, the present study proved for the first time that hUC-MSC transplantation has a good effect on both ACLI and ACLF rats. hUC-MSC transplantation can improve liver function, reduce liver injury/hepatocyte necrosis, and promote hepatocyte regeneration. hUC-MSCs may regulate the degree and progress of the immune response through paracrine mechanisms by upregulating HGF production to inhibit HSC activation, thereby interrupting the pathophysiological process of ACLI/ACLF, promoting the repair of liver tissue, and finally achieving the effect of treating ACLI/ACLF. And the downregulation on Notch, IFN-γ/Stat1, and IL-6/Stat3 signaling may contribute to the effect of hUC-MSCs in fibrosis inhibiting and hepatocyte regeneration. This study provided a theoretical basis and preclinical research evidence for the clinical application of hUC-MSC transplantation to treat ACLI and ACLF. However, due to the limited number of rats in this study, the long-term efficacy and the improvement in mortality due to hUC-MSC treatment for ACLF were not studied. Further study is required to evaluate the role of hUC-MSCs in decreasing the mortality of ACLF, and optimization of hUC-MSC treatment, especially in combination with agents targeting Notch and Stat1/Stat3 pathways.

## Supplementary Information

**Additional file 1: Figure S1.** The Phenotypes of hUC-MSCs. Flow cytometry analysis of hUC-MSCs for MSCs related markers. hUC-MSCs used for infusion were stained with CD105 (**a**), CD90 (**b**), CD73 (**c**), CD45 (**d**) and CD34 (**e**).

**Additional file 2: Table S1.** Primers for quantitative real-time PCR analysis.

**Additional file 3: Figure S2.** The integration of transplanted hUC-MSCs in the injured liver. Liver sections from ACLF and ACLI rats 24 hours post-hUC-MSC transplantation or 0.9% sodium chloride injection as a control were used for immunohistochemically staining of human-specific CD90.

**Additional file 4: Figure S3.** The expression of Notch signaling pathway related genes and Stat1/Stat3 signaling molecules in ACLI and ACLF rats. Total RNA and protein were extracted from the liver tissues from rats with ACLI or ACLF transplanted with hUC-MSCs or 0.9% sodium chloride as a control (*n* = 3). The mRNA expression levels of Notch1, Hes1, and P21 in ACLI rats were detected by quantitative PCR (**a**). Total protein was subjected to western blotting analysis. Relative protein expression of Stat1, pStat1, Stat3 and pStat3 in ACLF rats were quantitated (**b**). Relative protein expression of Stat1, pStat1, Stat3, pStat3, c-Myc, Bcl2 and CyclinD1 in ACLI rats were quantitated (**c-f**). Data were presented as the mean ± SD. *P < 0.05.

## Data Availability

The data supporting the conclusions of this article are available from the corresponding author upon request.

## Abbreviations

ACLF: Acute-on-chronic liver failure
ACLI: Acute-on-chronic liver injury
AFP: Alpha fetoprotein
ALB: Albumin
ALF: Acute liver failure
ALP: Alkaline phosphatase
ALT: Alanine aminotransferase
AST: Aspartate aminotransferase
CARS: Compensatory antiinflammatory response syndrome
CK18: Cytokeratin 18
CLD: Chronic liver disease
DBiL: Direct bilirubin
DCs: Dendritic cells
D-GalN: D-galactosamine
ECM: Extracellular matrix
FCS: Fetal calf serum
HA: Hyaluronic acid
HBV: Hepatitis B virus
HE: Hematoxylin-eosin
HGF: Hepatocyte growth factor
HSCs: Hepatic stellate cells
hUC-MSCs: Human umbilical cord-derived mesenchymal stem cells
IFN-γ: Interferon-γ
IL-1: Interleukin-1
IL-1β: Interleukin-1β
IL-1ra: Interleukin-1 receptor antagonist
IL-4: Interleukin-4
IL-6: Interleukin-6
IL-10: Interleukin-10
INR: International normalized ratio
LPS: Lipopolysaccharide
MELD: Model for end-stage liver disease
MMPs: Matrix metalloproteases
MMP9: Matrix metalloproteinase 9
MSCs: Mesenchymal stem cells
NAFLD: Nonalcoholic fatty liver disease
PCNA: Proliferating cell nuclear antigen
PGE2: Prostaglandin E2
PS: Porcine serum
PT: Prothrombin time
PIIINP: N-procollagen type III peptide
SD: Standard deviation
SI: Systemic inflammation
SIRS: Systemic inflammatory response syndrome
TBiL: Total bilirubin
TGF-β: Transforming growth factor-β
TGF-β1: Transforming growth factor-β1
TIMPs: Tissue inhibitors of matrix metalloproteases
TIMP1: Tissue inhibitor of metalloproteinase 1
TNF-α: Tumor necrosis factor-α
α-SMA: Alpha smooth muscle actin
