# Correction to: Human umbilical cord-derived mesenchymal stem cells improve the function of liver in rats with acute-on-chronic liver failure via downregulating Notch and Stat1/Stat3 signaling

**DOI:** 10.1186/s13287-022-02728-z

**Published:** 2022-02-07

**Authors:** Yulin He, Xingrong Guo, Tingyu Lan, Jianbo Xia, Jinsong Wang, Bei Li, Chunyan Peng, Yue Chen, Xiang Hu, Zhongji Meng

**Affiliations:** 1 Institute of Biomedical Research, Hubei Clinical Research Center for Precise Diagnosis and Treatment of Liver Cancer, Taihe Hospital, Hubei University of Medicine, Shiyan, 442000 Hubei China; 2 Hubei Key Laboratory of Embryonic Stem Cell Research, Shiyan, 442000 Hubei China; 3 Postgraduate Training Basement of Jinzhou Medical University, Taihe Hospital, Hubei University of Medicine, Shiyan, 442000 Hubei China; 4 Department of Infectious Diseases, Taihe Hospital, Hubei University of Medicine, Shiyan, 442000 Hubei China; 5 Department of Laboratory Medicine, Maternal and Child Health Hospital of Hubei Province, Wuhan, 430070 Hubei China; 6 Shenzhen Beike Biotechnology Research Institute, Nanshan District, Shenzhen, 518057 China

## Correction to: Stem Cell Research & Therapy (2021) 12:396 https://doi.org/10.1186/s13287-021-02468-6

The original article [1] contained incorrect affiliation numbers for each author which have since been corrected.
