# Supplementary figures and images for: Human umbilical cord-derived mesenchymal stem cells improve the function of liver in rats with acute-on-chronic liver failure via downregulating Notch and Stat1/Stat3 signaling
Source: Stem Cell Res Ther. 2021 Jul 13;12:396. doi: 10.1186/s13287-021-02468-6 (PMC8278604; doi:10.1186/s13287-021-02468-6)

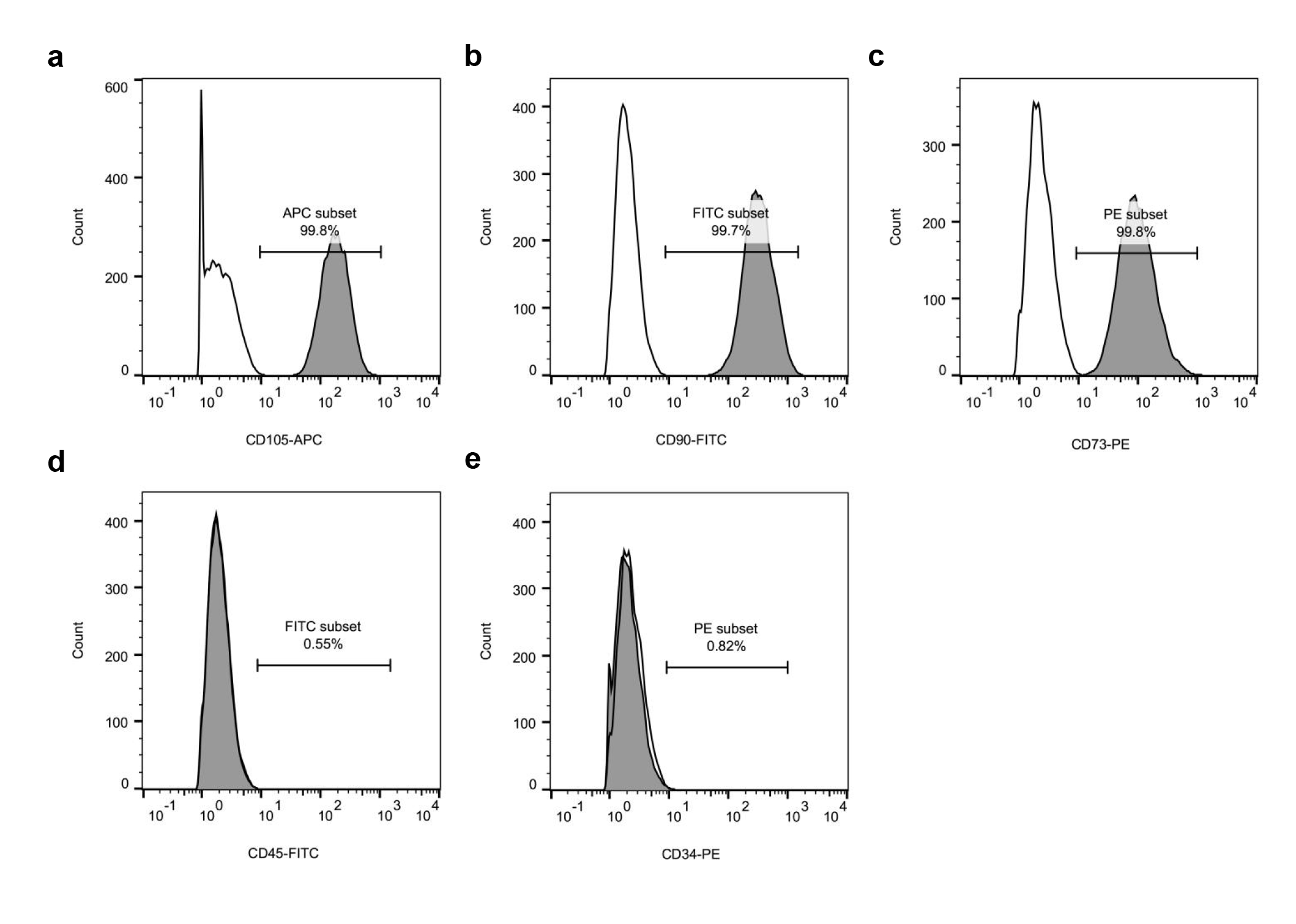

Supplement: Supplementary file 1 — Additional file 1: Figure S1. The Phenotypes of hUC-MSCs. Flow cytometry analysis of hUC-MSCs for MSCs related markers. hUC-MSCs used for infusion were stained with CD105 (a), CD90 (b), CD73 (c), CD45 (d) and CD34 (e). [file 13287_2021_2468_MOESM1_ESM.tif]

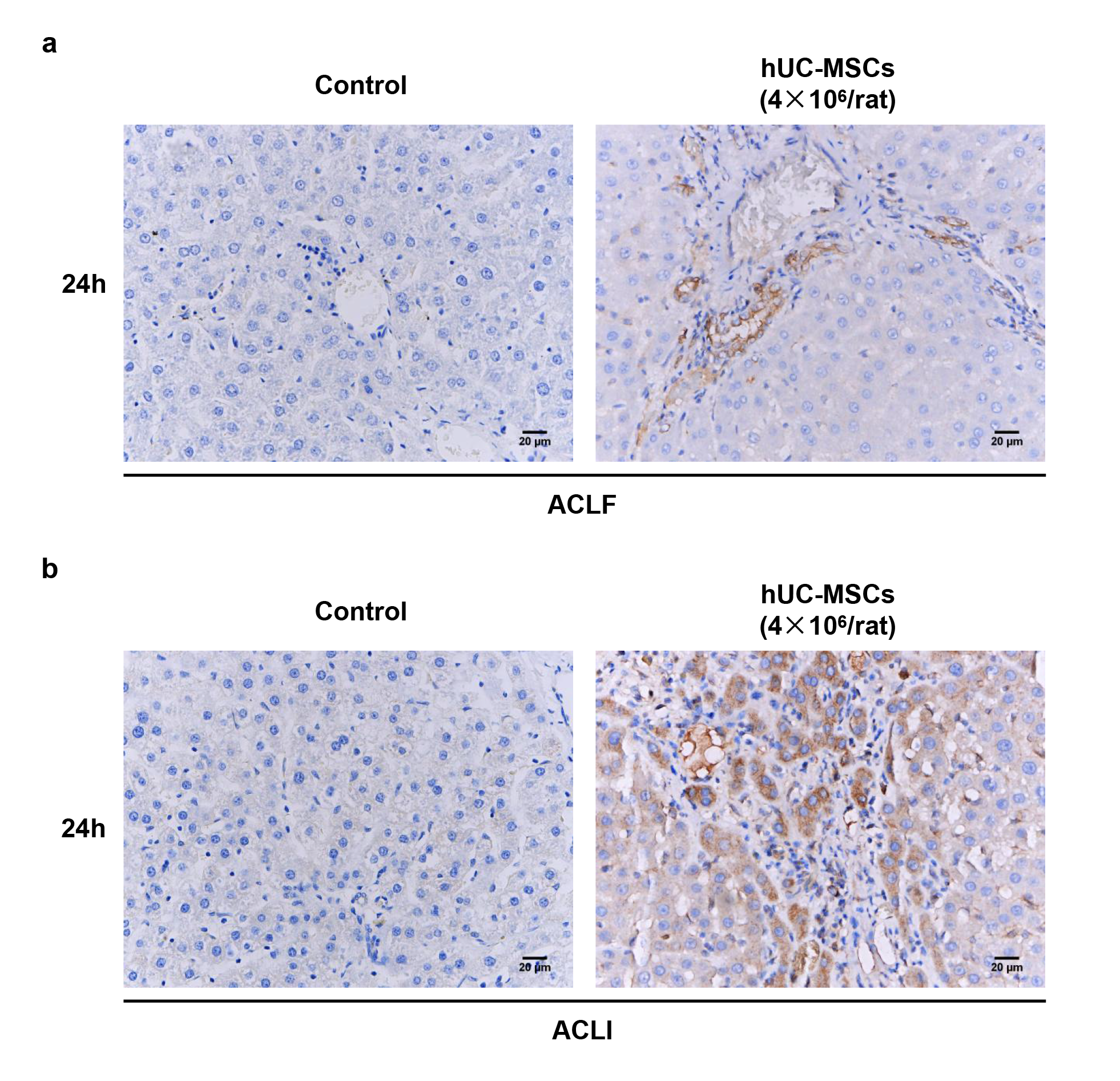

Supplement: Supplementary file 3 — Additional file 3: Figure S2. The integration of transplanted hUC-MSCs in the injured liver. Liver sections from ACLF and ACLI rats 24 hours post-hUC-MSC transplantation or 0.9% sodium chloride injection as a control were used for immunohistochemically staining of human-specific CD90. [file 13287_2021_2468_MOESM3_ESM.tif]

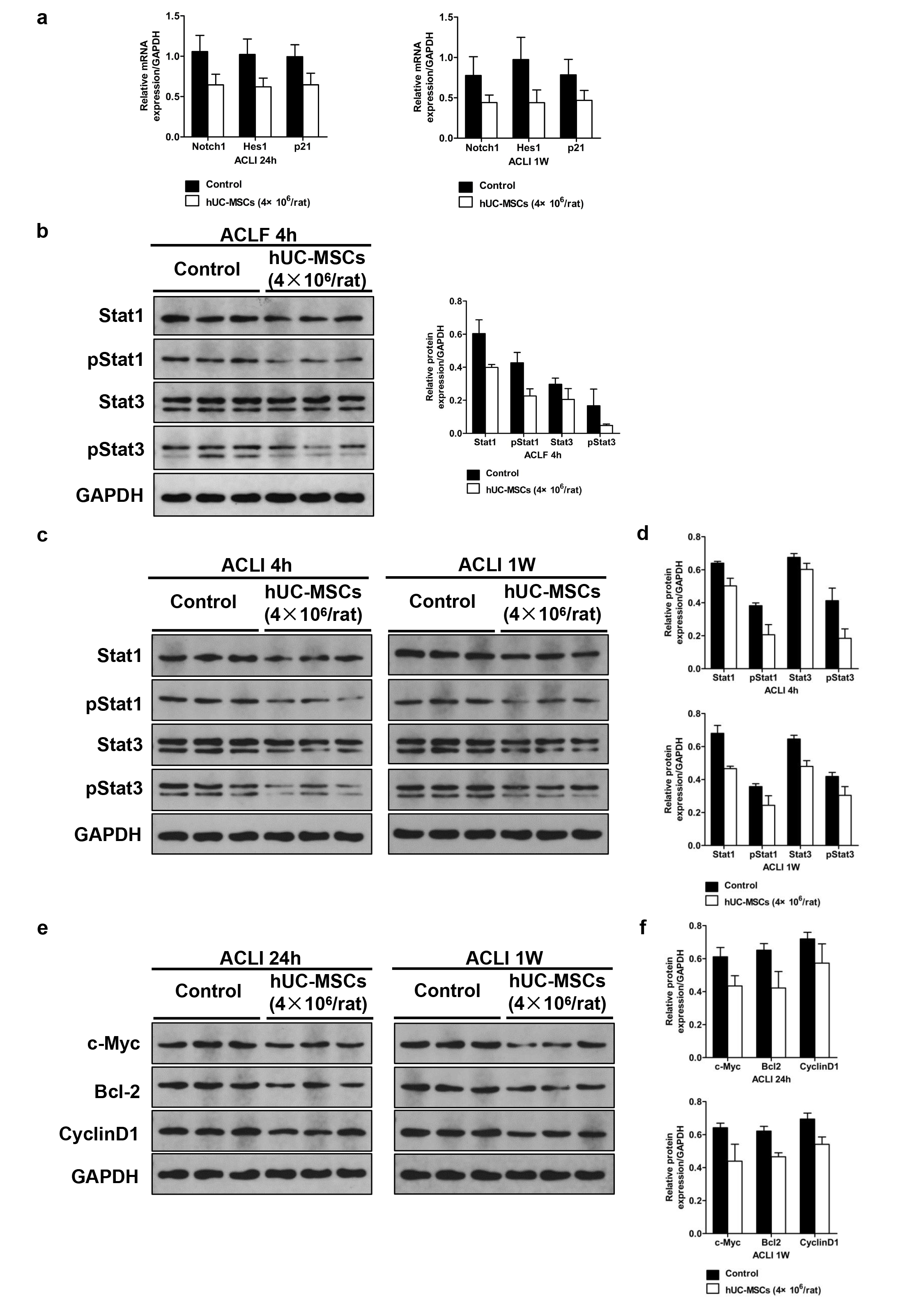

Supplement: Supplementary file 4 — Additional file 4: Figure S3. The expression of Notch signaling pathway related genes and Stat1/Stat3 signaling molecules in ACLI and ACLF rats. Total RNA and protein were extracted from the liver tissues from rats with ACLI or ACLF transplanted with hUC-MSCs or 0.9% sodium chloride as a control (n = 3). The mRNA expression levels of Notch1, Hes1, and P21 in ACLI rats were detected by quantitative PCR (a). Total protein was subjected to western blotting analysis. Relative protein expression of Stat1, pStat1, Stat3 and pStat3 in ACLF rats were quantitated (b). Relative protein expression of Stat1, pStat1, Stat3, pStat3, c-Myc, Bcl2 and CyclinD1 in ACLI rats were quantitated (c-f). Data were presented as the mean ± SD. *P < 0.05. [file 13287_2021_2468_MOESM4_ESM.tif]
