# Supplementary material for: Human umbilical cord-derived mesenchymal stem cells improve the function of liver in rats with acute-on-chronic liver failure via downregulating Notch and Stat1/Stat3 signaling
Source: Stem Cell Res Ther. 2021 Jul 13;12:396. doi: 10.1186/s13287-021-02468-6 (PMC8278604; doi:10.1186/s13287-021-02468-6)
Supplement: Supplementary file 2 — Additional file 2: Table S1. Primers for quantitative real-time PCR analysis. [file 13287_2021_2468_MOESM2_ESM.docx]

Table S1. Primers for quantitative real-time PCR analysis.

| Gene | Primer | |
| --- | --- | --- |
| GAPDH | F | ACAGCAACAGGGTGGTGGAC |
|  | R | TTTGAGGGTGCAGCGAACTT |
| Notch1 | F | CAGTACAACCCGCTAAGGC |
|  | R | GGACAAGGTATTGGTGGAGA |
| Hes1 | F | CAGGCTGGAGAGGCTGCCAAGGTTT |
|  | R | CCGAGGTCCCGCTGTTGCTGGTGTA |
| p21 | F | CAAAGTATGCCGTCGTCTGTT |
|  | R | AGTCAAAGTTCCACCGTTCTCG |
| c-Myc | F | CTGCTCTCCGTCCTATGTTGCG |
|  | R | TCAGGCTGGTGCTGTCTTTGCG |
| Bcl-2 | F | GCCTTCTTTGAGTTCGGTGG |
|  | R | CTGAGCAGCGTCTTCAGAGA |
| Cyclin D1 | F | GAGGAGCAGAAGTGCGAAGAGG |
|  | R | GGCGGATAGAGTTGTCAGTGTA |
